# Integrated Multi-Omics Analysis of Antarctic Krill Oil in Alleviating DSS-Induced Colitis and Modulating Gut Microbiota in Mice

**DOI:** 10.3390/md24080281

**Published:** 2026-08-14

**Authors:** Shuyin Yang, Xinnan Zhao, Tiantian Chen, Yichen Lin, Yan Di, Zhijun Tan, Ningning He, Shangyong Li, Jixing Peng

**Affiliations:** 1 School of Basic Medicine, Qingdao Medical College, Qingdao University, Qingdao 266071, China; syyang0312@163.com (S.Y.); lisy@qdu.edu.cn (S.L.); 2 Key Laboratory of Testing and Evaluation for Aquatic Product Safety and Quality, Ministry of Agriculture and Rural Affairs, Yellow Sea Fisheries Research Institute, Chinese Academy of Fishery Sciences, Qingdao 266071, China; zhaoxn@ysfri.ac.cn (X.Z.); diyan@ysfri.ac.cn (Y.D.);

**Keywords:** Antarctic krill oil, ulcerative colitis, PI3K-Akt signaling pathway, gut microbiota, multi-omics

## Abstract

Ulcerative colitis (UC) is a chronic inflammatory bowel disease with elusive etiology and limited therapeutic options, accompanied by potential side effects. In this study, to investigate the therapeutic potential of Antarctic krill oil (AKO) in a dextran sulfate sodium (DSS)-induced mouse colitis model, animal experiments, molecular assays, transcriptomics, metabolomics, and 16S rRNA gene sequencing were conducted. Our results revealed that supplementation of AKO significantly alleviated colitis symptoms, such as weight loss, and inflammatory responses. Moreover, multi-omics analyses demonstrated that AKO inhibited the PI3K/Akt signaling pathway, remodeled beneficial gut microbiota, and reshaped metabolite profiles associated with glycerolphospholipid metabolism. Remarkably, AKO alleviated DSS-induced colitis, accompanied by coordinated changes in the gut microbiota, metabolites, and transcriptome, which were associated with suppression of key inflammatory pathways. These findings present experimental evidence for the potential of AKO as a marine-based nutritional intervention for UC, and offer novel perspectives on microbiota-targeted therapies for inflammatory diseases.

## 1. Introduction

Ulcerative colitis (UC) and Crohn’s disease (CD) are the two principal subtypes of inflammatory bowel disease (IBD). UC is a chronic immune-mediated condition characterized by relapsing inflammation of the colonic mucosa, manifesting as chronic diarrhea, hematochezia, abdominal pain, and weight loss [1,2,3]. UC currently affects over 5 million people worldwide, with an annual incidence of 4.6–5.3 per 100,000 person-years, and its incidence continues to rise, particularly in newly industrialized regions [3,4,5].Current treatment aims to achieve sustained remission and mucosal healing, with standard therapies including corticosteroids, aminosalicylates, immunomodulators, and biologics [6]. However, prolonged use of these therapies may be associated with immune suppression and adverse effects [7,8]. Despite its unclear etiology, current evidence suggests that pathogenesis is linked to multiple interacting factors, including genetic predisposition, environmental factors, immune imbalance, and gut microbiota dysbiosis [9,10]. The gut microbiota is indispensable for intestinal homeostasis and contributes to metabolic, nutritional, and immune regulation. UC patients exhibit a characteristic decline in gut microbial diversity, reduced Firmicutes and elevated Proteobacteria, alongside compromised epithelial barrier integrity that enhances microbial translocation and inflammatory responses [11,12]. Based on these findings, microbiome-targeted therapies (MTTs) have emerged as promising alternatives to standard treatments [6]. In particular, naturally derived compounds with anti-inflammatory properties, such as n-3 polyunsaturated fatty acids (n-3 PUFAs), have gained attention for their capacity to modulate the gut microbiota and confer protective effects in IBD [13,14,15,16].

Antarctic krill oil (AKO), derived from the Antarctic-dwelling crustacean *Euphausia superba* [17], is abundant in bioavailable phospholipid-form n-3 PUFAs, especially docosahexaenoic acid (DHA) and eicosapentaenoic acid (EPA) [18], and also contains the antioxidant astaxanthin. Increasing evidence validates multiple health benefits of AKO. It has been reported to attenuate ethanol-induced acute gastric mucosal injury [19], improve symptoms and physical function in patients with knee osteoarthritis [20], reduce blood triglycerides (TGs) in hypertriglyceridemia individuals [21], and modulate cholesterol metabolism [22]. Additionally, AKO has shown neuroprotective effects through the microbiota–gut–brain axis and may alleviate cognitive dysfunction by suppressing oxidative stress and apoptosis in neurons [23,24]. Emerging evidence also points to the relevance of AKO in IBD. In a model of *Citrobacter rodentium*-induced infectious colitis, krill oil (KO) was shown to confer protection, along with increased abundance of butyrate-producing bacteria and improved microbial network interactions. In DSS-treated colitic mice, KO alleviated the UC symptoms via inhibiting the NF-κB pathway and modulating gut microbiota composition [25]. Furthermore, combined treatment with KO, *Lactobacillus reuteri*, and vitamin D was reported to significantly improve histological and clinical manifestations, promote mucosal healing, attenuate inflammatory responses, and modulate the gut microbiota [26].

Nevertheless, existing studies have predominantly adopted single-dimensional approaches, typically focusing on isolated biochemical indicators or specific metabolites. As a result, the mechanisms by which AKO ameliorates ulcerative colitis remain incompletely understood. In particular, the effects of AKO supplementation on host transcriptomic and fecal metabolomic profiles have not been fully characterized; moreover, the metabolic alterations induced by AKO in colitic mice have not been thoroughly investigated. To address this, we employed untargeted metabolomics to provide a comprehensive delineation of the metabolite profile changes. Furthermore, key regulatory pathways involved in AKO supplementation remain largely unexplored, and the role of the gut microbiota in AKO’s protective effects is still poorly defined as well. The integration of multi-omics would enable a more comprehensive understanding of the effects of AKO at different biological levels, thereby filling these critical research gaps.

In this study, we aimed to characterize the effects of AKO on host transcriptomic and fecal metabolomic profiles in DSS-induced colitis. Using an integrated multi-omics approach combining 16S rRNA sequencing, untargeted metabolomics, and transcriptomics, we identified a predominant enrichment of differential metabolites in glycerophospholipid metabolism, extending beyond short-chain fatty acids (SCFAs) [25]. In addition to the NF-κB pathway, which has been the focus of previous studies, our analysis suggested the involvement of the PI3K/Akt signaling pathway in AKO-mediated protection. These findings offer preliminary insights that may help inform future studies on AKO supplementation and its potential role in the dietary management of colitis.

## 2. Results

### 2.1. Supplementation of AKO Mitigated Clinical Manifestations of DSS-Induced Colitis in Mice

**Figure 1:**
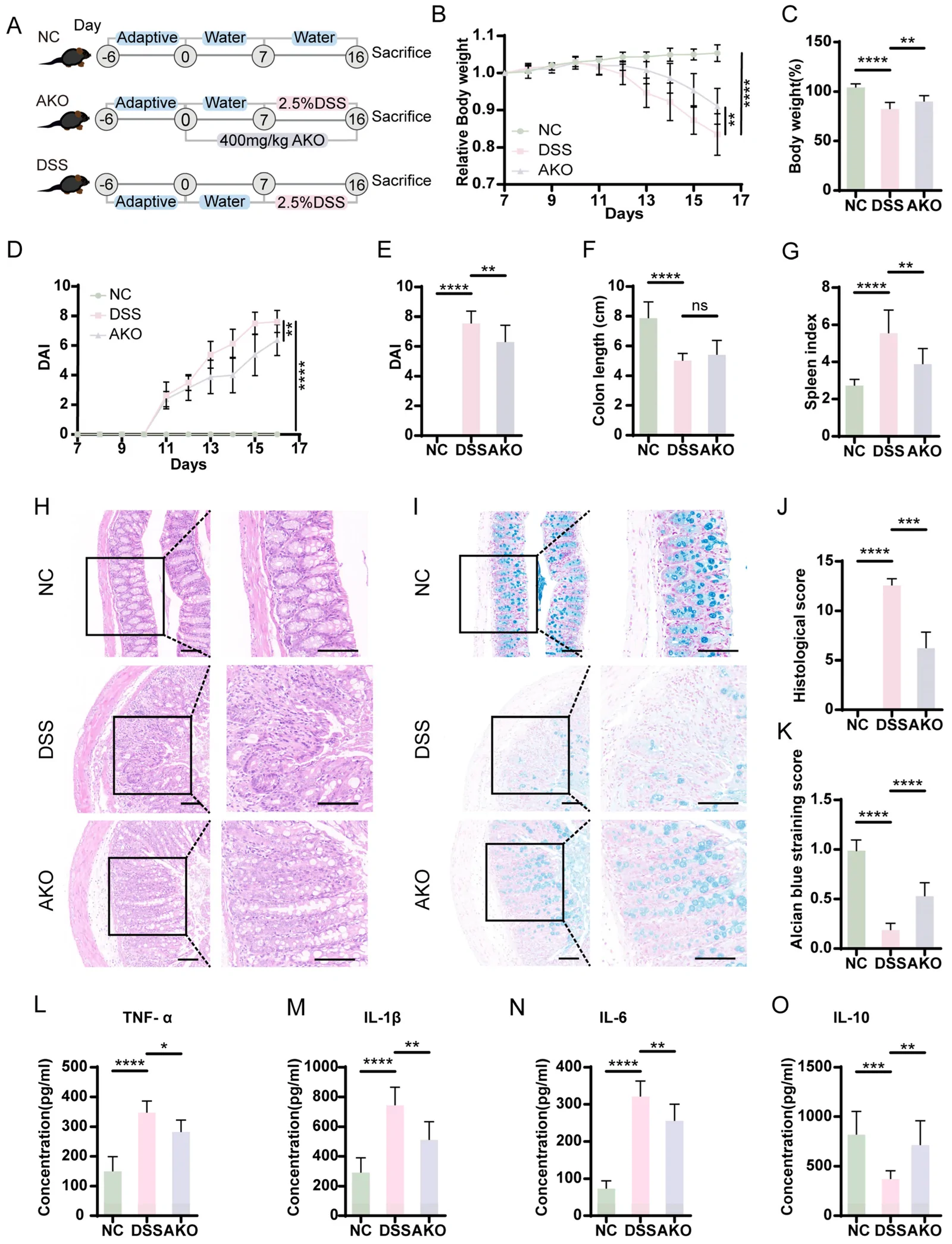
The supplementation of AKO markedly ameliorated the DSS-induced colitis in mice. (**A**) Experimental timeline. (**B**) Relative changes in body weight from the initial body weight. (**C**) Body weight was measured on the final experimental day. (**D**) The DAI index during the experiment. (**E**) DAI on the final experimental day. (**F**) Colon length and (**G**) spleen index in each group (n = 8). (**H**) Representative H&E-stained and (**I**) Alcian blue-stained colon sections. Scale bar: 100 μm. (**J**) histological scores; and (**K**) Alcian blue staining scores. (**L**–**O**) Serum levels of IL-1β, IL-6, IL-10, and TNF-α in each group. Data are presented as mean ± SD. Statistical significance is denoted as * *p* < 0.05, ** *p* < 0.01, *** *p* < 0.001, **** *p* < 0.0001, ns: not significant.

According to previous studies, the murine colitis model induced by DSS closely resembles the clinical manifestations of human UC [27]. Therefore, mice in the AKO group were prophylactically administered AKO for 1 week. From day 8 onward, 2.5% DSS solution was administered to induce the experimental model, while AKO supplementation was maintained continuously. The detailed experimental protocol is illustrated in Figure 1A.

Compared with the NC group, the DSS-treated mice’s body weight was markedly decreased, whereas AKO supplementation significantly alleviated this weight loss (Figure 1B,C). The severity of disease was further evaluated with the disease activity index (DAI) scores, which were notably lower in the AKO-treated group than in the DSS group (Figure 1D,E). DSS exposure also resulted in a marked reduction in colon length relative to the NC group. Although an increase in colon length was observed following AKO treatment compared with the disease model group, this change was not statistically significant (Figure 1F). Moreover, AKO supplementation significantly reduced the spleen index, an indicator of systemic inflammatory status [28] (Figure 1G).

Histological examination further substantiated the protective role of AKO. Hematoxylin and Eosin (H&E) staining and the corresponding histopathological scoring results demonstrated that the DSS group exhibited pronounced glandular destruction, crypt loss, inflammatory cell infiltration, goblet cell depletion, and mucosal epithelial necrosis, whereas these pathological abnormalities were improved by the AKO treatment (Figure 1H,J). Consistently, Alcian blue staining (Figure 1I) and corresponding scores (Figure 1K) demonstrated a marked decline in mucin synthesis in the DSS group, while AKO intervention partially restored mucin secretion.

In addition, serum levels of inflammatory cytokines were assessed by Enzyme-linked immunosorbent assay (ELISA). Relative to the NC group, DSS exposure disrupted the inflammatory cytokine balance, as reflected by markedly elevated IL-1β, IL-6, and TNF-α levels and reduced IL-10 levels (Figure 1L–O). Collectively, these results indicate that AKO supplementation can mitigate DSS-induced colitis by attenuating intestinal inflammation and maintaining mucosal barrier function.

### 2.2. AKO Supplementation Ameliorated Colitis in Mice Through Colonic Transcriptome Reprogramming, Especially via the PI3K-Akt Signaling Pathway

**Figure 2:**
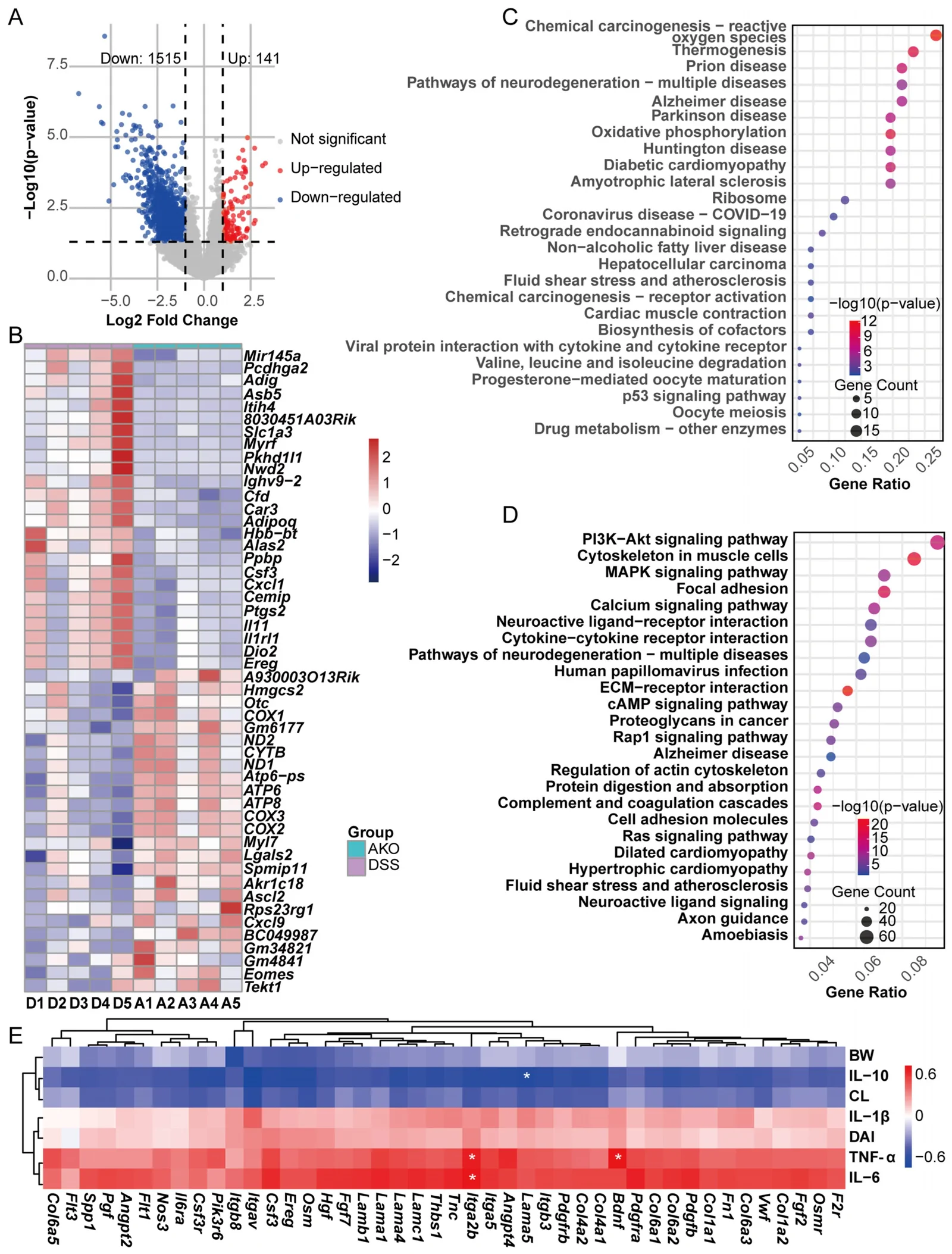
AKO-mediated remodeling of the colonic transcriptome and marked inhibition of PI3K/Akt signaling in DSS-treated mice. (n = 5) (**A**) Volcano plot of DEGs between the AKO and DSS groups. (**B**) Heatmap of the top 25 upregulated and top 25 downregulated DEGs. (**C**,**D**) KEGG enrichment of (**C**) upregulated and (**D**) downregulated DEGs. (**E**) Heatmap of Pearson correlations between phenotypic indicators and PI3K/Akt pathway-related DEGs. * *p* < 0.05.

To explore the potential mechanisms through which AKO ameliorates DSS-induced colitis, transcriptomic analysis was conducted on colon tissues from experimental mice. Differentially expressed genes (DEGs) altered by AKO were identified with the DESeq2 package (v 1.50.2). DEGs were screened with thresholds of |log_2_ (fold change)| ≥ 1 and *p* < 0.05. Based on these thresholds, the AKO group exhibited 1515 downregulated and 141 upregulated genes relative to the DSS group (Figure 2A). Subsequently, we constructed a heatmap (Figure 2B) to visualize the distinct gene expression profiles among the groups, highlighting AKO’s regulatory effects on the intestinal transcriptome. To further characterize the biological pathways involved in AKO-mediated protection, Kyoto Encyclopedia of Genes and Genomes (KEGG) enrichment analysis was then conducted for all DEGs. The enriched pathways were ranked by gene ratio and separately visualized for upregulated and downregulated DEGs using bubble plots (Figure 2C,D). Among the pathways enriched by downregulated DEGs, PI3K/Akt signaling showed significant enrichment and included the highest gene count (n = 66), suggesting that this pathway may be involved in the protective actions of AKO.

DEG analysis between the NC and DSS groups selected 1683 upregulated and 412 downregulated genes in the DSS group (Figure S1A). A total of 729 DSS-dysregulated genes were reversed by AKO treatment (Figure S1B). Integrated analysis of these two comparisons identified 44 genes in the PI3K/Akt pathway. Pearson analysis was performed between these 44 genes and colitis-associated phenotypic indices, including colon length (CL), body weight (BW), DAI, TNF-α, IL-6, IL-1β, and IL-10. Heatmap visualization showed negative correlations of these DEGs with CL, BW, and IL-10, but positive correlations with DAI and the pro-inflammatory cytokines. To further examine the interactions among these genes, they were uploaded to the STRING database for protein–protein interaction analysis (PPI). Hub genes within the candidate gene set were identified by Maximal Clique Centrality (MCC) analysis using the CytoHubba plugin in Cytoscape 3.10.3, followed by construction of the PPI network. With a threshold of MCC > 1 × 10^10^, a total of 20 genes were ultimately screened, such as *Col1a1*, *Itgb3*, *Itga5*, *Col6a1*, *Col4a1*, and *Col4a2* (Figure S1C).

In summary, our research found that AKO ameliorates experimental colitis by inhibiting the PI3K/Akt pathway-related gene expression.

### 2.3. AKO Supplementation Regulated Gut Metabolite Profiles in Mice with DSS-Induced Colitis

**Figure 3:**
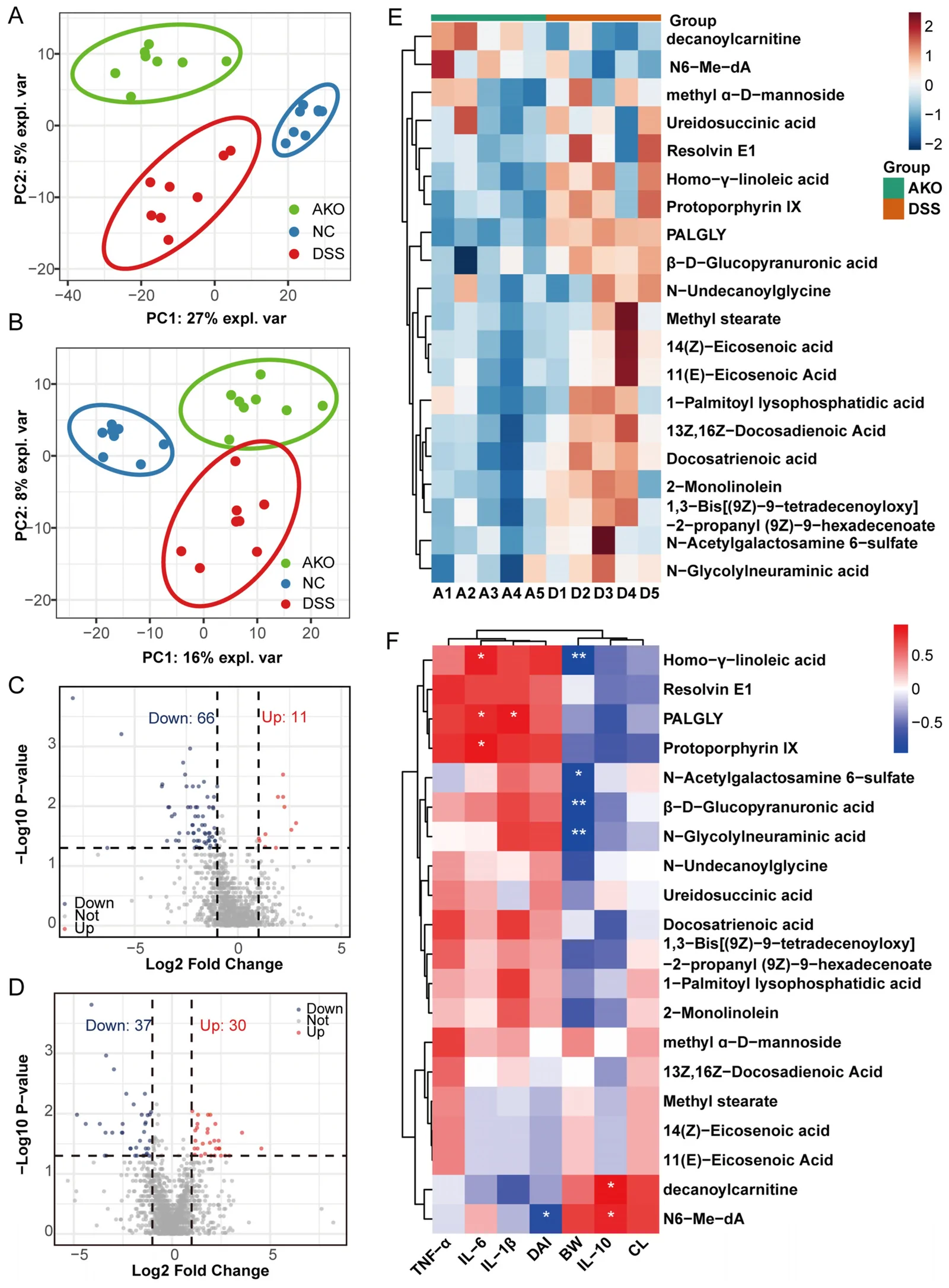
AKO-induced reshaping of fecal metabolite profiles with prominent effects on lipid metabolism in DSS-induced colitis mice. (**A**,**B**) PLS-DA of fecal metabolites in (**A**) negative and (**B**) positive ion modes (n = 8). (**C**,**D**) Volcano plots of differentially expressed metabolites between the AKO and DSS groups in (**C**) negative and (**D**) positive ion modes. (**E**) Heatmap of differential metabolites between the AKO and DSS groups (n = 5). (**F**) Heatmap of Pearson correlations between differential metabolites and phenotypic indicators. * *p* < 0.05, ** *p* < 0.01.

Published evidence suggests that alterations in intestinal metabolite composition and abundance can directly or indirectly influence the intestinal transcriptome, and AKO has been shown to promote SCFA biosynthesis [29,30]. Untargeted metabolomics was performed to elucidate the mechanisms by which AKO supplementation alleviates the colitis induced by DSS. Partial least squares discriminant analysis (PLS-DA) in both negative-ion mode (Figure 3A) and positive-ion mode (Figure 3B) showed tight clustering within groups and clear separation among groups. Notably, the DSS group was clearly distinct from the NC and AKO groups, suggesting that AKO supplementation markedly shifts gut metabolite profiles.

Furthermore, differential metabolite identification was conducted, and the results were visualized utilizing volcano plots with screening criteria of |log_2_ Fold Change| ≥ 1 and *p* < 0.05. Compared to the DSS group, 66 downregulated and 11 upregulated metabolites were selected in negative-ion mode (Figure 3C), whereas 37 downregulated and 30 upregulated metabolites were detected in positive-ion mode (Figure 3D). Relative to the NC group, DSS altered 261 metabolites (152 upregulated and 109 downregulated) in negative-ion mode and 530 metabolites (163 upregulated and 367 downregulated) in positive-ion mode (Figure S2A,B). After excluding artificial compounds, 20 metabolites dysregulated by DSS and restored by AKO were retained for further analysis. A clustered heatmap (Figure 3E) showed that two metabolites (decanoylcarnitine and N6-Me-dA) were upregulated, while the other 18 metabolites were downregulated.

To further examine the potential relevance of these metabolites to colitis severity, the 20 differential metabolites were correlated with colitis-related phenotypic indices using Pearson’s method (Figure 3F). Homo-γ-linoleic acid, PALGLY, and Protoporphyrin IX exhibited marked positive correlations with the pro-inflammatory cytokine IL-6, and PALGLY was also correlated with IL-1β. In contrast, the upregulated metabolites N6-Me-dA and decanoylcarnitine showed significant positive correlations with IL-10. N6-Me-dA also showed a marked negative correlation with DAI. Moreover, Homo-γ-linoleic acid, β-D-Glucopyranuronic acid, N-Acetyl galactosamine 6-sulfate, and N-Glycolylneuraminic acid were significantly negatively correlated with body weight. Enrichment analysis of these 20 compounds through MetaboAnalyst linked them mainly to Glycerolipid Metabolism and Phospholipid Biosynthesis (Figure S2C,D).

Taken together, our analyses reveal that AKO supplementation markedly reshapes the perturbed fecal metabolome in DSS-induced colitic mice, with the restored metabolites mainly enriched in glycerolipid metabolism and phospholipid biosynthesis pathways.

### 2.4. AKO Supplementation Reshaped Gut Microbiota Composition in DSS-Induced Colitis Mice

Extensive evidence indicates that alterations in gut microbiota composition are closely linked to the development and progression of UC [31,32]. In UC patients, dysbiosis is generally manifested by reduced microbial diversity, depletion of beneficial bacteria, and enrichment of potentially pathogenic taxa, which together contribute to disease aggravation [33]. 16S rRNA gene sequencing of fecal samples was conducted to assess the regulatory effect of AKO supplementation on gut microbiota in colitic mice.

**Figure 4:**
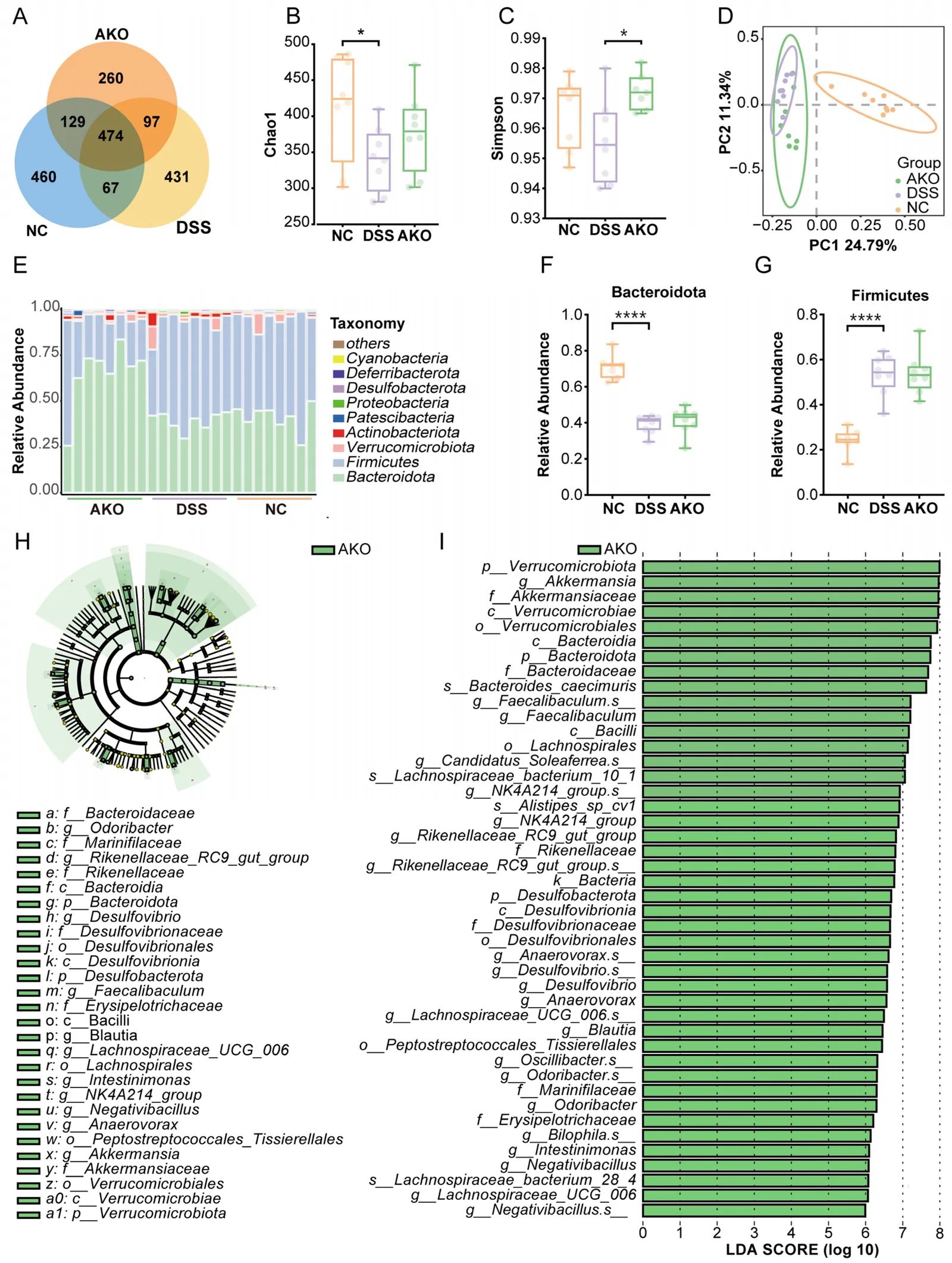
Partial restoration of gut microbiota following AKO supplementation in DSS-induced colitis mice. (n = 8) (**A**) Venn diagram of OTUs identified by 16S rRNA gene sequencing. (**B**,**C**) Alpha diversity indices in different groups, including (**B**) Chao1 and (**C**) Simpson indices. (**D**) PCoA plot of microbial community structure. (**E**) Phylum-level relative abundance of gut microbiota. (**F**,**G**) Relative abundance of (**F**) Bacteroidota and (**G**) Firmicutes at the phylum level. (**H**,**I**) (**H**) Cladogram of taxa identified by LEfSe analysis; (**I**) corresponding histogram. Taxa at multiple taxonomic ranks, including species (s), genera (g), families (f), orders (o), classes (c), and phyla (p), were identified as differentially abundant when *p* < 0.15 and LDA score (log_10_) > 6. * *p* < 0.05, **** *p* < 0.0001.

The Venn diagram showed 460, 431, and 260 group-specific OTUs in the NC, DSS, and AKO groups, respectively, with 474 OTUs shared among all three groups and 129 OTUs shared between the NC and AKO groups. These observations indicated that AKO intervention partially reshaped the DSS-disrupted gut microbiota profile (Figure 4A). For alpha diversity, Chao1 and Shannon indices were markedly reduced in the DSS group relative to the NC group, whereas no notable differences were observed between the AKO and DSS groups (Figure 4B and Figure S3A). The Simpson index was significantly higher in the AKO group than in the DSS group, whereas the NC and DSS groups did not differ significantly (Figure 4C). Principal Coordinates Analysis (PCoA) was implemented to evaluate intergroup beta diversity, revealing a distinct separation in gut microbiota composition among groups. The microbial profiles of the DSS and AKO groups were distinct from those of the NC group, although partial overlap remained between the DSS and AKO groups (Figure 4D).

Analysis of relative taxonomic abundance indicated that Bacteroidota and Firmicutes constituted the predominant phyla in all groups (Figure 4E and Figure S3B). DSS exposure significantly decreased the relative abundance of Bacteroidota and increased Firmicutes abundance relative to the NC group, whereas no notable differences were observed between the DSS and AKO groups (Figure 4F,G). Additionally, AKO supplementation led to a significant reduction in Actinobacteriota abundance, while Proteobacteria were significantly enriched in the DSS group. No significant alterations were detected for the remaining phyla (Figure S3C–G). Linear discriminant analysis effect size (LEfSe) revealed 44 differential taxa, all of which were exclusively enriched in the AKO group, including *Akkermansia*, *Bacteroides*, *Faecalibaculum*, *Lachnospiraceae*, and other taxa (Figure 4H,I). These taxa may serve as potential microbial biomarkers associated with AKO-mediated improvement of colitis.

**Figure 5:**
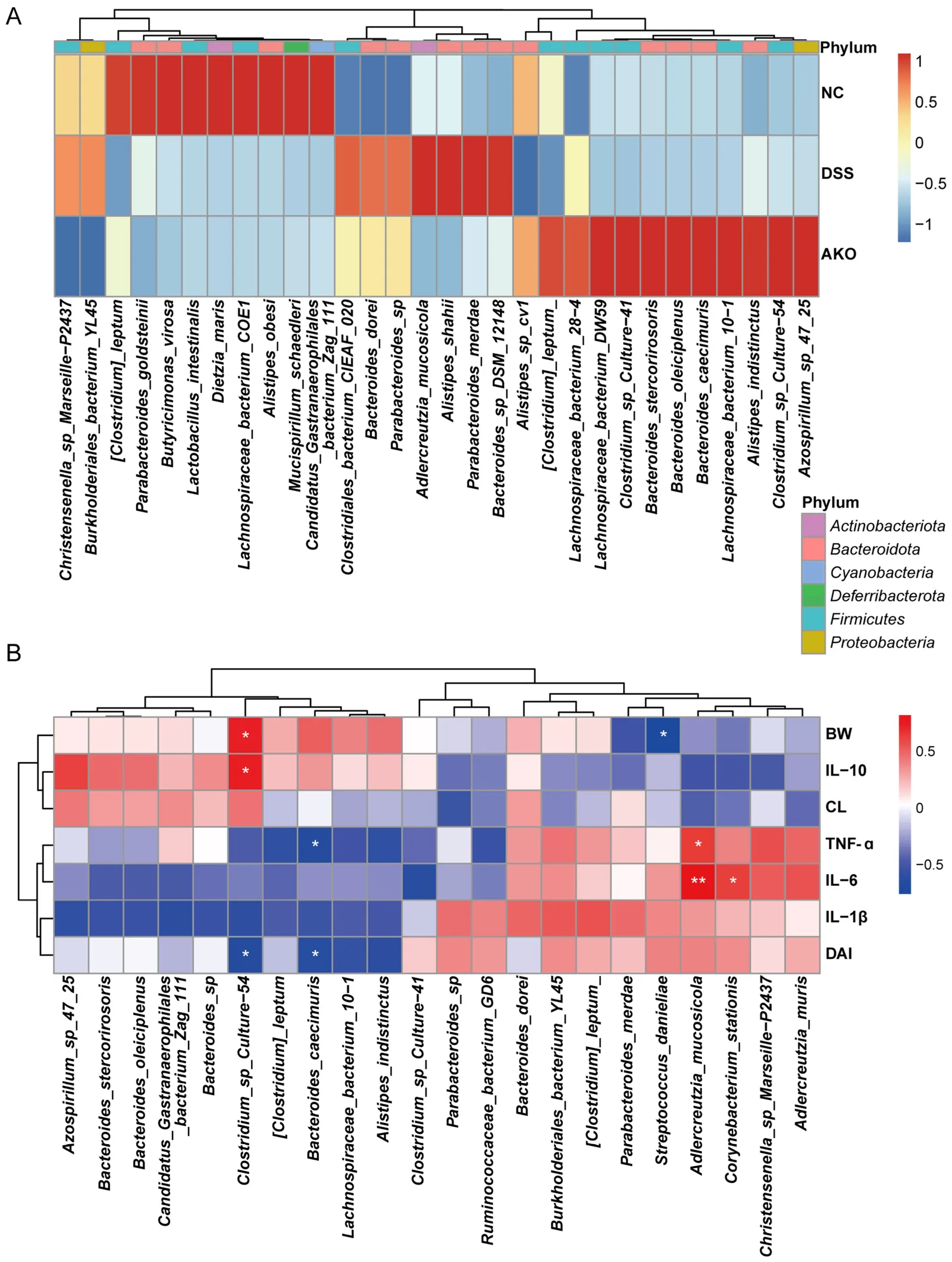
AKO-induced alterations in gut microbial species composition. (**A**) Heatmap of intergroup differences in the top differentially abundant bacterial species. (**B**) Heatmap of Pearson correlations between gut microbial taxa and phenotypic indicators. (|r| > 0.5). * *p* < 0.05, ** *p* < 0.01.

Enrichment analysis of differentially abundant taxa was employed at the species, genus, and family levels (Figure 5A and Figure S3H,I). The heatmap showed marked differences in species-level composition among groups, with 12 significantly enriched species, including *Lachnospiraceae_bacterium_28-4*, *Lachnospiraceae_bacterium_DW59*, *Clostridium_sp_Culture-41*, *Bacteroides_stercorirosoris*, *Bacteroides_caecimuris*, *Clostridium_sp_Culture-54*, and others.

Furthermore, Pearson correlation analysis of enriched species and phenotypic indices further supported their potential relevance to disease severity (Figure 5B). *Clostridium_sp_Culture-54* exhibited a significantly positive correlation with body weight and IL-10 levels, whereas *Clostridium_sp_Culture-54* and *Bacteroides_caecimuris* were significantly inversely associated with DAI. *Bacteroides_caecimuris* was also significantly and inversely correlated with TNF-α. However, *Adlercreutzia_mucosicola* showed significantly positive correlations with pro-inflammatory cytokines TNF-α and IL-6, and *Corynebacterium_stationis* was significantly positively correlated with IL-6. *Streptococcus_danieliae* was significantly inversely correlated with body weight.

Collectively, these findings suggest that AKO supplementation ameliorates colitis through modulating gut microbial composition and enriching beneficial microbial taxa.

### 2.5. Integrated Multi-Omics Analysis Uncovered the Potential Mechanisms of AKO Supplementation Alleviating DSS-Induced Ulcerative Colitis in Mice

**Figure 6:**
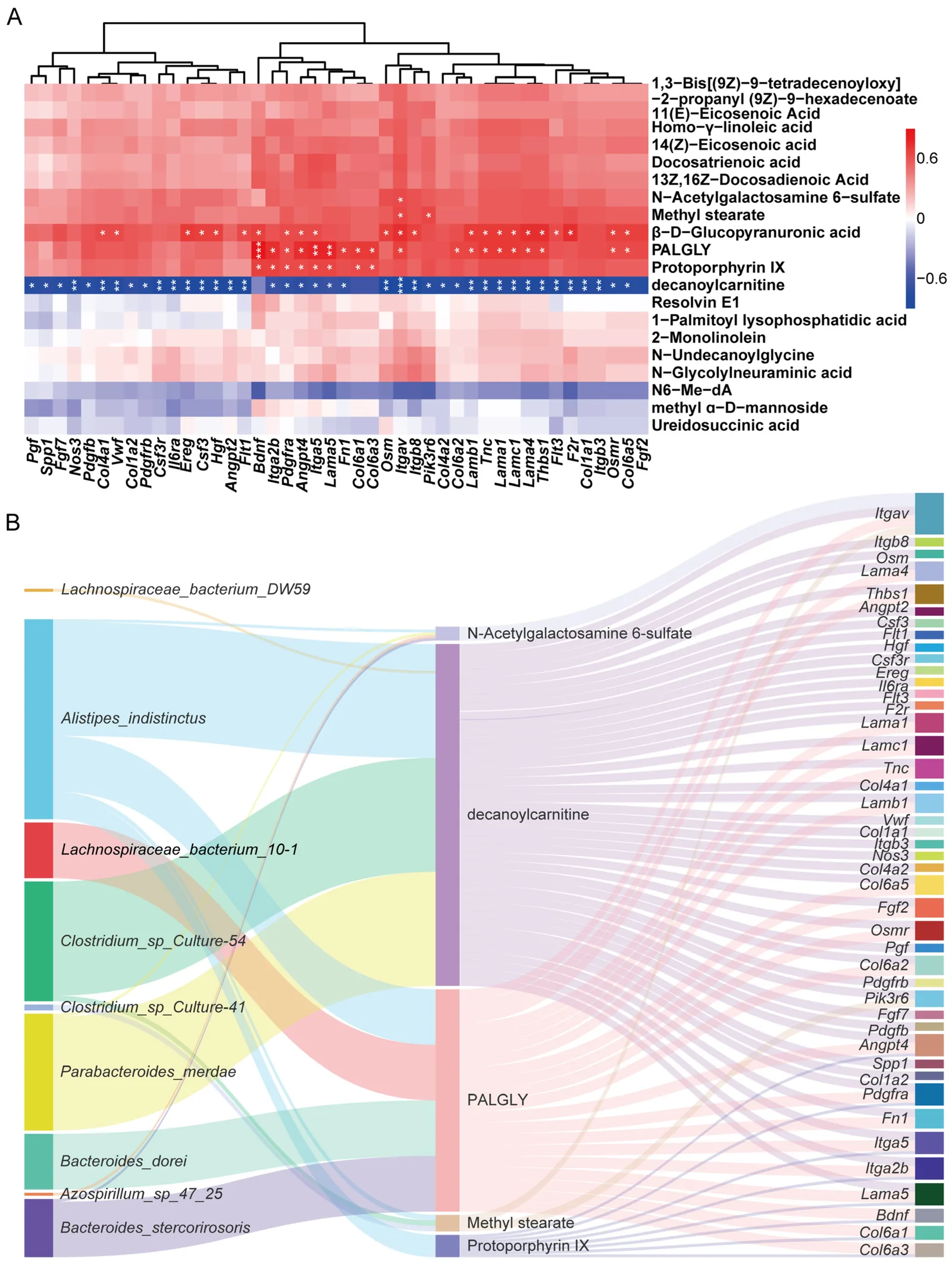
Integrated multi-omics analysis of gut microbiota, metabolites, and PI3K/Akt pathway-related DEGs. (**A**) Heatmap showing Spearman correlations between PI3K/Akt pathway-related DEGs and differential metabolites, * *p* < 0.05, ** *p* < 0.01, *** *p* < 0.001. (**B**) Sankey diagram depicting the regulatory interactions among microbial taxa, differential metabolites, and PI3K/Akt pathway-related DEGs. |r| ≥ 0.5 and *p* < 0.05.

Transcriptomic, metabolomic, and microbiota analyses were performed separately, confirming that AKO mitigated DSS-induced colitis in mice by attenuating the PI3K/Akt signaling pathway, reshaping fecal metabolite profiles related to lipid metabolism, and modulating gut microbial dysbiosis. However, the interactions among these three layers remained unclear. To further investigate their potential crosstalk, Spearman correlation analysis was performed between PI3K/Akt-related DEGs and metabolites, as well as between metabolites and microbial taxa (Figure 6A and Figure S4).

As expected, the upregulated metabolite decanoylcarnitine was significantly negatively correlated with most of the DEGs, while downregulated metabolites N-Acetylgalactosamine 6-sulfate, β-D-Glucopyranuronic acid, PALGLY, Protoporphyrin IX and 13Z,16Z-Docosadienoic Acid were significantly positively correlated with these genes.

Additionally, the AKO-enriched taxa generally showed positive associations with upregulated metabolites and negative associations with downregulated metabolites. *Bacteroides_dorei* was significantly positively correlated with PALGLY, 13Z,16Z-Docosadienoic Acid, Docosatrienoic acid, 2-Monolinolein and 1-Palmitoyl lysophosphatidic acid. *Parabacteroides merdae* showed positive correlations with β-D-Glucopyranuronic acid, 1-Palmitoyl lysophosphatidic acid, 2-Monolinolein, Docosatrienoic acid, 13Z,16Z-Docosadienoic Acid, N-Acetylgalactosamine 6-sulfate, 1,3-Bis[(9Z)-9-tetradecenoyloxy]-2-propanyl (9Z)-9-hexadecenoate and N-Glycolylneuraminic acid, but was significantly inversely correlated with decanoylcarnitine. *Parabacteroides_sp* showed a significant positive correlation with N-Glycolylneuraminic acid, whereas *Alistipes_sp_cv1* and *Azospirillum_sp_47_25* showed a significantly negative correlation with N-Acetylgalactosamine 6-sulfate.

Notably, *Clostridium_sp_Culture-54* showed significantly negative correlations with downregulated metabolites, including N-Glycolylneuraminic acid, N-Undecanoylglycine, 13Z,16Z-Docosadienoic Acid, Docosatrienoic acid, 1,3-Bis[(9Z)-9-tetradecenoyloxy]-2-propanyl (9Z)-9-hexadecenoate, 11(E)-Eicosenoic Acid, 14(Z)-Eicosenoic acid, Homo-γ-linolenic acid, Methyl stearate and β-D-Glucopyranuronic acid, whereas it showed significant positive correlations with up-regulated metabolites. Similarly, *Clostridium_sp_Culture-41* was significantly negatively correlated with 13Z,16Z-Docosadienoic acid, Docosatrienoic acid, 1,3-Bis[(9Z)-9-tetradecenoyloxy]-2-propanyl (9Z)-9-hexadecenoate, 2-Monolinolein, 1-Palmitoyl lysophosphatidic acid, 11(E)-Eicosenoic acid, 14(Z)-Eicosenoic acid, Homo-γ-linolenic acid, Methyl stearate and β-D-Glucopyranuronic acid. *Alistipes_indistinctus* was significantly negatively correlated with Homo-γ-linolenic acid, Methyl stearate, β-D-Glucopyranuronic acid, PALGLY, Protoporphyrin IX, N-Acetylgalactosamine 6-sulfate, and 13Z,16Z-Docosadienoic Acid, but positively correlated with decanoylcarnitine. In addition, *Lachnospiraceae_bacterium_DW59* showed significantly negative correlations with 2-Monolinolein and N-Acetylgalactosamine 6-sulfate. Both *Bacteroides_oleipelogenes* and *Bacteroides_stercoris* were significantly negatively correlated with N-Acetylgalactosamine 6-sulfate and PALGLY. *Lachnospiraceae_bacterium_28_4* demonstrated significant negative correlations with 13Z,16Z-Docosadienoic Acid, Docosatrienoic acid, 1,3-Bis[(9Z)-9-tetradecenoyloxy]-2-propanyl (9Z)-9-hexadecenoate, 2-Monolinolein, 1-Palmitoyl lysophosphatidic acid. *[Clostridium]_leptum* was significantly negatively correlated with 13Z,16Z-Docosadienoic Acid, methyl α-D-mannoside, 1,3-Bis[(9Z)-9-tetradecenoyloxy]-2-propanyl (9Z)-9-hexadecenoate, 2-Monolinolein and 1-Palmitoyl lysophosphatidic acid. *Lachnospiraceae_bacterium_10-1* demonstrated significant negative correlations with PALGLY, 13Z,16Z-Docosadienoic Acid, Docosatrienoic acid, 1-Palmitoyl lysophosphatidic acid, 14(Z)-Eicosenoic acid, and Homo-γ-linolenic acid.

To further elucidate the underlying mechanism, gut microbiota, metabolites, and PI3K/Akt pathway-related DEGs were integrated for multi-omics analysis, and their associations were visualized in a Sankey diagram. (Figure 6B). Several key microbial taxa including *Lachnospiraceae_bacterium_DW59*, *Alistipes_indistinctus*, *Lachnospiraceae_bacterium_10-1*, *Clostridium_sp_Culture-54*, *Clostridium_sp_Culture-41*, *Parabacteroides_merdae*, *Bacteroides_dorei*, *Azospirillum_sp_47_25* and *Bacteroides_stercorirosoris* were significantly associated with metabolites such as N-Acetyl galactosamine 6-sulfate, decanoylcarnitine, PALGLY, methyl stearate and Protoporphyrin IX, which were in turn closely linked to the DEGs. These results highlighted a core microbiota-metabolite-transcriptome axis, suggesting that AKO alleviates colitis by modulating gut microbial composition, reshaping metabolite profiles, and consequently suppressing PI3K/Akt signaling.

**Figure 7:**
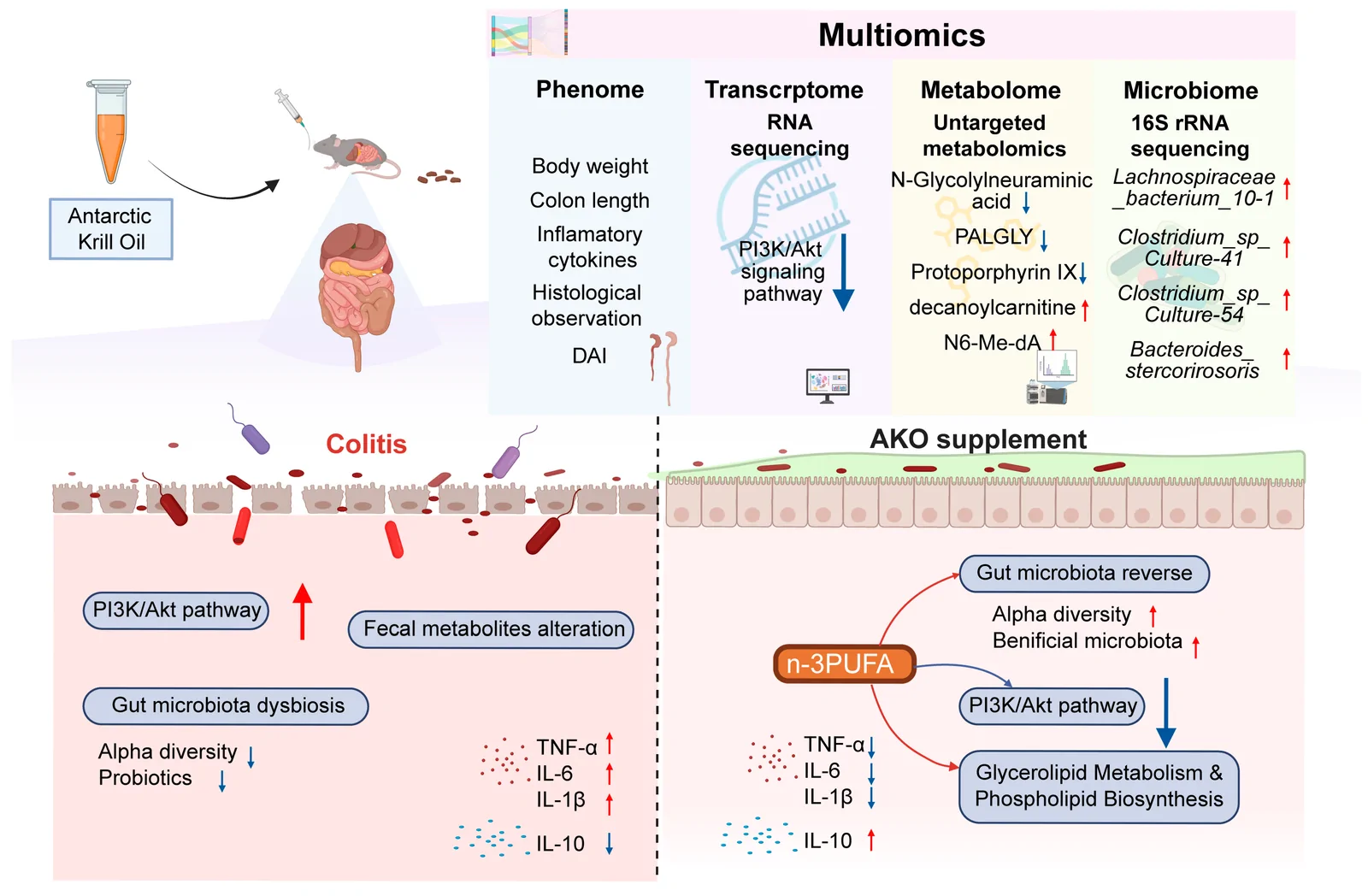
The potential mechanism of AKO alleviating UC.

Overall, our study, which combined animal experiments, ELISA assays, and multi-omics analyses, demonstrates that AKO effectively alleviates DSS-induced colitis in mice (Figure 7). AKO preserved intestinal epithelial integrity, inhibited the PI3K/Akt signaling pathway, regulated glycerolipid metabolism and phospholipid biosynthesis, and restored gut microbiota homeostasis. Collectively, these results reveal that AKO confers protection against colitis through coordinated modulation of the transcriptome, gut microbiota, and metabolome.

## 3. Discussion

UC is a chronic immune-mediated disorder characterized by recurrent mucosal inflammation, with symptoms including diarrhea, hematochezia, weight loss, and abdominal pain [1,2,3]. UC currently affects over 5 million people worldwide, with its incidence continuing to rise globally [3]. Its etiology is generally attributed to interactions among environmental factors, genetic susceptibility, immune dysregulation, and gut microbial imbalance [9,10]. The long-term use of conventional therapies such as corticosteroids, aminosalicylates, immunomodulators, and biologics is often limited by adverse effects and safety risks. These limitations have driven growing interest in safe, microbiome-targeted nutritional interventions as complementary strategies for UC management [13,14].

AKO is a marine-derived lipid obtained from *Euphausia superba* [17], and contains abundant n-3 PUFAs. Unlike many other omega-3 sources, these fatty acids in AKO are predominantly esterified to phospholipids, which confer higher bioavailability [18]. Accumulating evidence indicates that n-3 PUFAs confer protection against colitis by strengthening intestinal barrier function, preserving epithelial integrity, regulating butyrate-producing taxa, and suppressing the NF-κB and MAPK signaling pathways that drive intestinal inflammation [34,35,36]. Additionally, in the present work, we established a DSS-induced colitis model to assess the protective role of AKO. To gain further insight into the mechanisms involved, we integrated transcriptomic, metabolomic, and gut microbiota analyses, with particular emphasis on the crosstalk among these three biological layers.

Our findings showed that the AKO treatment significantly ameliorated DSS-induced colitis. Specifically, AKO attenuated body weight loss, relieved clinical symptoms, and improved mucosal injury. In addition, it suppressed pro-inflammatory cytokine production (TNF-α, IL-1β, IL-6) while enhancing the level of the anti-inflammatory cytokine IL-10.

The PI3K/Akt pathway is involved in multiple biological processes, including cell proliferation, differentiation, metabolism, and survival [37]. Disruption of this pathway has been implicated in various pathological conditions, such as metabolic syndrome, gastrointestinal disorders, and intestinal inflammation [38,39]. Several studies have illustrated that *Lacticaseibacillus casei 39* [40], *Bifidobacterium longum BAA-2573* [41] and *Bacteroides cellulosilyticus* [42] can alleviate colitis through suppressing the PI3K/Akt signaling pathway. Similarly, DHA has also been reported to exert protective effects against colitis through inhibiting this pathway [43]. In contrast, previous studies have reported that *Clostridium butyricum* and *Christensenellaceae minuta* exert protective effects on intestinal barrier function in colitis through activation of PI3K/Akt signaling [44,45]. In another study, coadministration of *Lactobacillus* and *Bifidobacterium* strains was found to initiate signaling pathways associated with epithelial barrier protection [46,47].

Previous studies have reported integrin β3 upregulation in UC patients and experimental colitis models [48]. The ER stress-related genes *COL1A1* and *VWF* have also been shown to be positively correlated with UC inflammation [49]. ColVI is not only elevated in DSS colitis and restored upon DSS withdrawal, but also plays a critical role in the progression and resolution of intestinal inflammation by modulating lymphangiogenesis, lymphatic drainage, and the induction of pro-inflammatory cytokines [50,51]. *Col4A1* is upregulated in both UC and CD and has been identified as a key diagnostic signature gene for UC. It encodes the most abundant component of the basement membrane and may potentially contribute to intestinal mucosal fibrosis [52,53].

Furthermore, our transcriptomic results revealed that AKO supplementation significantly suppressed the PI3K/Akt signaling pathway, which contributed to rebalancing of inflammatory cytokine profiles, restoration of intestinal epithelial integrity and subsequent attenuation of inflammation (Figure 2A–D). Existing literature has reported that modulation of PI3K/Akt signaling regulates the downstream targets, thereby promoting intestinal barrier restoration and suppressing pro-inflammatory cytokine production [54,55]. In line with previous studies, our correlation analysis further linked PI3K/Akt-related gene expression to inflammatory cytokine alterations. By preserving intestinal barrier function, AKO may reduce mucosal immune exposure to luminal antigens and thereby limit aberrant activation of inflammatory signaling pathways [54,56].

Disruption of gut microbial homeostasis is considered an important factor involved in UC pathogenesis. It can increase mucosal permeability, facilitate the translocation of bacterial toxins, and promote immune dysregulation, thereby forming a vicious cycle that aggravates inflammation [57,58]. In contrast, beneficial commensals can suppress inflammatory activity [59].

Growing evidence suggests that n-3 PUFAs can favorably modulate gut microbiota composition. For instance, fish oil enriches *Bifidobacterium* and *Roseburia*, two genera that are inversely associated with UC development [60,61]. Fish oil supplementation in lactating women also increases the DHA content of breast milk, which increases the abundance of *Bifidobacterium* and *Lactobacillus* in infant feces [62]. In addition, DHA-PL and EPA-PL ameliorate microbiota dysbiosis through promoting *Bifidobacteria* growth, suppressing *Enterobacteria* proliferation, thus abrogating inflammation associated with intestinal dysfunction [63]. AKO has also been shown to alter gut microbiota composition through increasing the abundance of Firmicutes while reducing that of Bacteroidetes at the phylum level, and restoring several key genera, including *Bacteroides*, *Paraprevotella*, *the [Eubacterium] nodatum group*, and the *Rikenellaceae RC9 gut group* [25].

In line with these observations, our results further revealed that AKO supplementation enriched a number of specific taxa at the species level, such as *Clostridium* sp., *Bacteroides caecimuris*, and *Lachnospiraceae_bacterium_28-4*. Notably, several of these AKO-enriched taxa have been previously linked to beneficial host effects. Clostridium sp. intervention attenuates inflammation and hippocampal neuronal injury and reverses statin-induced glucose intolerance in mice [32,33]. *Lachnospiraceae_bacterium_28-4* and *Lachnospiraceae_bacterium_DW59* may be closely associated with the anti-obesity effects of naringin, by stimulating adipose thermogenesis and browning [34]. *Bacteroides_caecimuris* has also been reported to augment immune responses in fungal infection models as well as in murine ileocecal resection models, potentially through regulation of immune cell differentiation and cytokine secretion [35,36]. In our study, the relative abundances of these taxa inversely correlated with DAI and pro-inflammatory cytokines, whereas positive associations were observed with body weight and IL-10 (Figure 4 and Figure S3). One exception was *Alistipes_indistinctus*, which was previously reported to be reduced in colitic mice following heat-killed *Bifidobacterium bifidum HB1628* treatment [37], but showed a different trend in our study, warranting further investigation. Collectively, these findings suggest that AKO protects against colitis by restoring gut microbial homeostasis and alleviating dysbiosis.

Beyond the gut microbiota, the fecal metabolite alterations observed in our study also merit attention, and several of the restored metabolites have been previously documented. In our work, untargeted metabolomic analysis identified 20 differential metabolites, most of which were lipid-related. Differential metabolites have been implicated in the regulation of inflammatory processes. Several of these metabolites have been previously implicated in the regulation of inflammatory processes. For instance, PALGLY promotes NO production through activation of calcium-sensitive nitric oxide synthase enzymes such as endothelial nitric oxide synthase (eNOS), whose activation is closely linked to the PI3K/Akt signaling pathway [29]. Another study reported that zinc protoporphyrin IX aggravated DSS-induced colitis, as reflected by higher DAI scores and increased colonic TNF-α and IFN-γ levels, which supports our results [30]. Additionally, upregulation of N-glycolylneuraminic acid was reversed after Chicoric acid supplementation in colitic mice [31]. In contrast, existing literature suggests that Resolvin E1, an EPA-derived metabolite, can prevent the development of DSS-induced colitis in mice [32], and garcinoic acid was shown to increase Resolvin E1 levels during colitis [33]. However, the relative abundance of Resolvin E1 was decreased in our study, suggesting that the underlying mechanism requires further investigation. Beyond these metabolites, existing studies have demonstrated that AKO supplementation can reshape fecal metabolite profiles, including increasing SCFA levels [25,36]. Specifically, AKO supplementation has been reported to enrich butyrate-producing bacteria and elevate butyrate abundance [25,30]. In a separate study, butyrate supplementation in DSS-induced colitic mice was shown to suppress the PI3K/Akt pathway and promote autophagy in intestinal epithelial cells, thereby alleviating colitis [64].

Furthermore, enrichment analysis highlighted that these metabolites were mainly involved in glycerolipid metabolism and phospholipid biosynthesis, indicating that AKO extensively remodels lipid metabolism in colitis mice. Previous studies have shown that phospholipids can serve as precursors for the construction and repair of intestinal epithelial cell membranes, thereby enhancing barrier integrity [65]. Beyond this structural role, glycerophospholipid metabolism has been identified as a key pathway dysregulated in DSS-induced colitis. HLJDD was shown to alleviate colitis by inhibiting COX-2, PLA_2_, and 5-LOX, thereby restoring glycerophospholipid and arachidonic acid metabolism [66]. SYD similarly modulated glycerophospholipid metabolism and unsaturated fatty acid biosynthesis via inhibiting the PI3K/Akt-mTOR pathway [67]. Furthermore, TFA was reported to correct glycerophospholipid metabolic disorders, thereby inhibiting M1 macrophage polarization and alleviating UC [68]. These findings collectively suggest that AKO-derived phospholipids support epithelial membrane repair while restoring glycerophospholipid homeostasis to attenuate macrophage-driven inflammation.

Integrated multi-omics analysis further revealed strong correlations among key gut microbial taxa, differential metabolites, and PI3K/Akt-related DEGs. In this study, AKO supplementation modulated gut microbiota composition, reshaped metabolic profiles, and suppressed the PI3K/Akt pathway. These alterations were interrelated, but the causal directionality among them remains to be elucidated. Rather than a single linear mechanism, our findings point to a complex, interconnected multi-omics network that potentially underlies the beneficial effects of AKO.

In comparison to fish oil, AKO contains relatively lower n-3 PUFA levels, but shows higher bioavailability owing to its phospholipid-bound fatty acids [18]. Moreover, as a marine product derived from a lower trophic level, AKO carries a lower risk of bioaccumulating harmful substances and thus offers potential safety advantages [69,70]. Although AKO, DHA, EPA, and fish oil all exert anti-inflammatory effects, they may differ in their regulation of specific microbial species and metabolites, which may have implications for personalized nutritional interventions [71,72]. However, direct comparisons remain difficult because of differences in experimental design, dosage, and intervention conditions, and further standardized studies are needed.

This work offers mechanistic insights into how AKO ameliorates DSS-induced colitis, with emphasis on the interactions among gut microbiota, metabolites, and inflammatory signaling pathways. Our findings suggest that AKO may ameliorate experimental colitis through preserving intestinal epithelial integrity, partially correcting gut microbial dysbiosis, reshaping metabolite profiles, and modulating inflammation-related signaling. These findings extend current understanding of the role of n-3 PUFAs in IBD intervention through gut microbiota regulation and provide additional molecular and microbiological evidence for the source-specific functional properties of n-3 PUFA supplements. AKO is already marketed as a dietary supplement and has demonstrated good tolerability in several human trials, such as knee osteoarthritis and cardiovascular disease [73,74,75]. Combined with its phospholipid-associated bioavailability, this safety profile suggests that AKO may offer advantages over conventional fish oil as a nutritional intervention.

Furthermore, the dose used in this study (400 mg/kg/day in mice) corresponds to a human equivalent dose of approximately 1.94 g/day for a 60 kg adult [76]. This dose falls within the typical krill oil supplementation dose [74,75] and is substantially higher than the generally insufficient daily dietary intake of marine n-3 PUFAs in most populations, suggesting supplementation is necessary to achieve the observed benefits [77]. These characteristics support the translational potential of AKO as a feasible nutritional intervention for IBD. By integrating gut microbiota, metabolomic, and transcriptomic analyses, this study provides a useful framework for future studies into the gut microecological actions of n-3 PUFA supplementation. The distinct gut microbes and metabolites associated with different n-3 PUFA supplements may also offer clues for the development of more individualized nutritional strategies for IBD management.

However, several limitations should be acknowledged. First, the key regulatory relationships among specific microbial taxa, metabolites, and the PI3K/Akt pathway were inferred primarily from correlation analyses and therefore require further experimental validation, such as Western blotting, qPCR, and in vitro functional assays. Second, only a single dose of AKO was tested, and the dose–effect relationship warrants further investigation with multiple dose levels (e.g., 100, 200, and 400 mg/kg). Third, the present findings are derived from a DSS-induced colitis mouse model, which does not fully recapitulate the heterogeneity and chronicity of human UC. In addition, inter-individual variability in gut microbiota composition and metabolic response to n-3 PUFAs may affect the reproducibility of these multi-omics benefits in human populations. While existing clinical trials have demonstrated good short-term tolerability of AKO, the long-term safety of sustained supplementation, particularly regarding oxidative stability, contaminant exposure, and drug interactions, has not been established for UC patients. Future studies should validate the causal relationships, determine the optimal dose, and evaluate the long-term efficacy and safety of AKO in clinical settings.

In conclusion, AKO may serve as a promising functional supplement for adjuvant UC therapy and may provide a basis for the development of microbiome-targeted nutritional strategies for IBD.

## 4. Materials and Methods

### 4.1. Antarctic Krill Oil

AKO was obtained from the Yellow Sea Fisheries Research Institute. A total of 236 lipid species were identified in AKO samples. In positive ion mode, 221 lipid species were detected, including acylcarnitines (AcCa), ceramides (Cer), coenzyme Q10 (CoQ10), diacylglycerol phosphates (DAP), lysophosphatidylethanolamines (LPE), phosphatidylcholines (PC), phosphatidylethanolamines (PE), triglycerides (TG), and diglycerides (DG). In negative ion mode, 22 compounds were identified, including PC, lysophosphatidylcholines (LPC), and PE. Fatty acid analysis showed that saturated fatty acids (SFAs), monounsaturated fatty acids (MUFAs), and polyunsaturated fatty acids (PUFAs) accounted for 38.7%, 17.0%, and 44.2%, respectively. DHA and EPA accounted for 15.9% and 15.6% of total fatty acids, respectively. PC constituted approximately 37.5% of AKO. The detailed compositional profile of AKO is shown in Figure S5.

### 4.2. Animal Experiments and Sample Preparation

Male C57BL/6J mice aged 6 weeks and weighing 18–22 g (SPF grade) were purchased from Pengyue (Jinan, China). Animals were housed four per cage in an SPF-grade facility under controlled conditions, with a 12 h light/dark cycle, relative humidity maintained at 50 ± 15%, and ambient temperature kept at 22 ± 2 °C. Following one week of acclimation, mice were randomly allocated to three groups, with eight animals in each group. According to the experimental protocol, the mice received regular drinking water or 2.5% DSS solution ad libitum. The groups were treated as follows: NC group: no treatment; DSS group: mice were orally gavaged with 0.1 mL distilled water for 16 days and received 2.5% DSS solution from day 8 to day 16; AKO group: mice were orally gavaged with Antarctic krill oil at 400 mg/kg/day for 16 days and received 2.5% DSS solution from day 8 to day 16. Body weight was measured and recorded on a daily basis. This dose was chosen based on the effective range (250–500 mg/kg/day) established in the same model [25]. In order to assess the severity of intestinal inflammation, the DAI was determined using criteria including body weight loss, stool consistency, and the presence of fecal blood. For body weight loss assessment, a 5% reduction relative to the initial body weight was assigned 1 point, with a maximum cumulative score of 3. Stool consistency was scored as follows: 0, normal consistency; 1, loose stools; and 2, diarrhea. Fecal blood was scored as follows: 0, no bleeding; 1–2, detectable bleeding; and 3, conspicuous bleeding. The single-blind design was adopted to evaluate the therapeutic efficacy among groups. For subsequent analysis of gut microbiota composition and metabolomic profile, before the end of the experiment, fecal samples were collected and stored at −80 °C.

After completion of the treatment regimen, all mice were anesthetized with isoflurane, and blood samples were collected via retro-orbital bleeding, after which the mice were euthanized by cervical dislocation. Serum was isolated by centrifugation at 3000 rpm for 15 min at 4 °C, then stored at −80 °C for inflammatory cytokine measurement. For histological evaluation, a mid-colon segment was preserved in 4% paraformaldehyde, while an adjacent segment was snap-frozen in liquid nitrogen for RNA sequencing. The spleens were excised for gravimetric analysis and evaluation of the spleen index [28].

### 4.3. Enzyme-Linked Immunosorbent Assay

The inflammatory cytokine levels of serum samples, including IL-6, IL-1β, TNF-α, and IL-10, were measured via ELISA kits (Abclonal, Wuhan, China) in line with protocols.

### 4.4. Histological Examination

Histopathological alterations in colon tissues were examined via H&E staining, and Alcian blue staining was utilized to measure mucin production. For more detailed methods, please refer to our previous studies [78,79].

### 4.5. Gut Microbiota Analysis

The V3–V4 region of bacterial 16S rRNA genes was amplified by polymerase chain reaction (PCR), followed by sequencing library construction and amplicon sequencing on the NovaSeq 6000 platform (Shenzhen, Guangdong, China). Sequence data were processed with QIIME2, and valid reads were clustered into OTUs at a 97% similarity threshold. The sequence with the highest abundance within each OTU was retained as the representative sequence and used for subsequent analyses on the NovoMagic cloud platform (Shenzhen, Guangdong, China) [80,81].

### 4.6. Transcriptome Analysis

We commissioned Berry Genomics Co., Ltd. (Beijing, China) to perform transcriptome sequencing. Total RNA was extracted from 150 mg of colon tissue using an RNA extraction kit (AC0202; Sparkjade Biotech, Jinan, China) followed by assessment of RNA purity and concentration with an Agilent 2100 Bioanalyzer (Agilent Technologies, Santa Clara, CA, USA). cDNA libraries were then prepared from qualified RNA samples, and library quality was evaluated utilizing the Agilent 2100 DNA Bioanalyzer and the Quant-iT PicoGreen dsDNA Assay Kit (Thermo Fisher Scientific, Waltham, MA, USA). The libraries were sequenced on the Illumina Genome Analyzer platform. Raw data were processed using R software (v4.5.1). KEGG pathway enrichment of the DEGs was analyzed using the clusterProfiler package (v4.18.4). Furthermore, PPI networks were established based on the STRING database, and hub genes were identified and visualized with Cytoscape 3.10.3. More detailed methods have been described in our previous study [79,80].

### 4.7. Untargeted Metabolomic Analysis

Fecal samples were prepared as follows. 100 mg colon contents were homogenized in 1.2 mL of methanol/acetonitrile/water (2:2:1, *v*/*v*/*v*), and then vortexed for 30 s. The homogenates were subjected to sonication at 4 °C for 10 min, followed by snap-freezing in liquid nitrogen and incubation at −20 °C for 1 h to precipitate proteins. Following centrifugation at 13,000 rpm for 15 min at 4 °C, 1 mL of the supernatant was aspirated and dried with a vacuum concentrator. Finally, the dried extracts were redissolved in 100 μL of acetonitrile/water (1:1, *v*/*v*), yielding the final solutions for subsequent LC–MS analysis. A 10 μL aliquot from each sample was then combined to prepare quality control (QC) samples [82]. Pooled QC samples were injected at regular intervals, and features with RSD > 30% were excluded.

Metabolite profiling was conducted by a Thermo Scientific UPLC-T3–Q Exactive Orbitrap MS system (Waltham, MA, USA) equipped with a heated electrospray ionization (HESI) source. Data acquisition was carried out in both positive and negative ESI modes to enable comprehensive analyte detection. Detailed instrumental parameters and detection conditions have been elaborated in our previous research [80].

Raw data were acquired and processed using Compound Discoverer (v 3.1) for preliminary metabolite identification based on mass-to-charge ratio (*m*/*z*) and retention time. Metabolite annotation was performed using a self-constructed metabolite database established by the Yellow Sea Fisheries Research Institute. Metabolic pathways were identified via MetaboAnalyst.

### 4.8. Statistical Analysis

Graphical visualization and statistical analysis were performed using GraphPad Prism 10.2.1. Differences between two groups were evaluated using Student’s *t*-test or the Mann–Whitney U test, as appropriate, whereas comparisons among multiple groups were performed using one-way ANOVA or the Kruskal–Wallis test.

The R Stats package (R version 4.5.1) was used for RNA-seq transcriptome analysis, pathway enrichment analysis, and untargeted metabolomic analysis. Some bioinformatic analyses were performed and visualized by the OECloud tools [83]. The Sankey diagram was generated with the OmicShare tool [84]. The mechanism diagram was created by BioRender.com and Adobe Illustrator 2024.

Experimental data are presented as the mean ± standard deviation (SD). Associations between variables were determined utilizing Spearman correlation analysis or Pearson correlation analysis, with the threshold for statistical significance set at *p* < 0.05. Detailed bioinformatics and statistical methods were as previously described [42,80,85].

## 5. Conclusions

This study showed that AKO effectively mitigated DSS-induced colitis in mice through preserved intestinal epithelial integrity, regulated cytokine balance, reshaped gut microbiota composition, altered metabolite profiles, and modulated inflammation-related signaling. Multi-omics analyses suggested that AKO’s benefits may arise from multi-layered interactions rather than a single linear mechanism. These results deepen current understanding of the mechanisms underlying AKO-mediated protection against colitis and may inform the development of future nutritional intervention strategies for UC.

## 6. Patents

The authors declare no related patents.

## Data Availability

All data included in this work are available upon request.

## Supplementary Materials

The following supporting information can be downloaded at: https://www.mdpi.com/article/10.3390/md24080281/s1, Figure S1: Transcriptomic profiling and protein–protein interaction analysis of DEGs; Figure S2: Metabolomic profiling and pathway enrichment analysis of differential metabolites; Figure S3: 16S rRNA analysis of gut microbial community composition; Figure S4: (A) Spearman correlation heatmap between the top 20 bacterial species and differential metabolites; Figure S5: Composition of AKO.
