# Supplementary material for: Integrated Multi-Omics Analysis of Antarctic Krill Oil in Alleviating DSS-Induced Colitis and Modulating Gut Microbiota in Mice
Source: Mar Drugs. 2026 Aug 14;24(8):281. doi: 10.3390/md24080281 (PMC13514700; doi:10.3390/md24080281)
Supplement: Supplementary file 1 [file marinedrugs-24-00281-s001.zip › marinedrugs-4450911-supplementary.pdf]

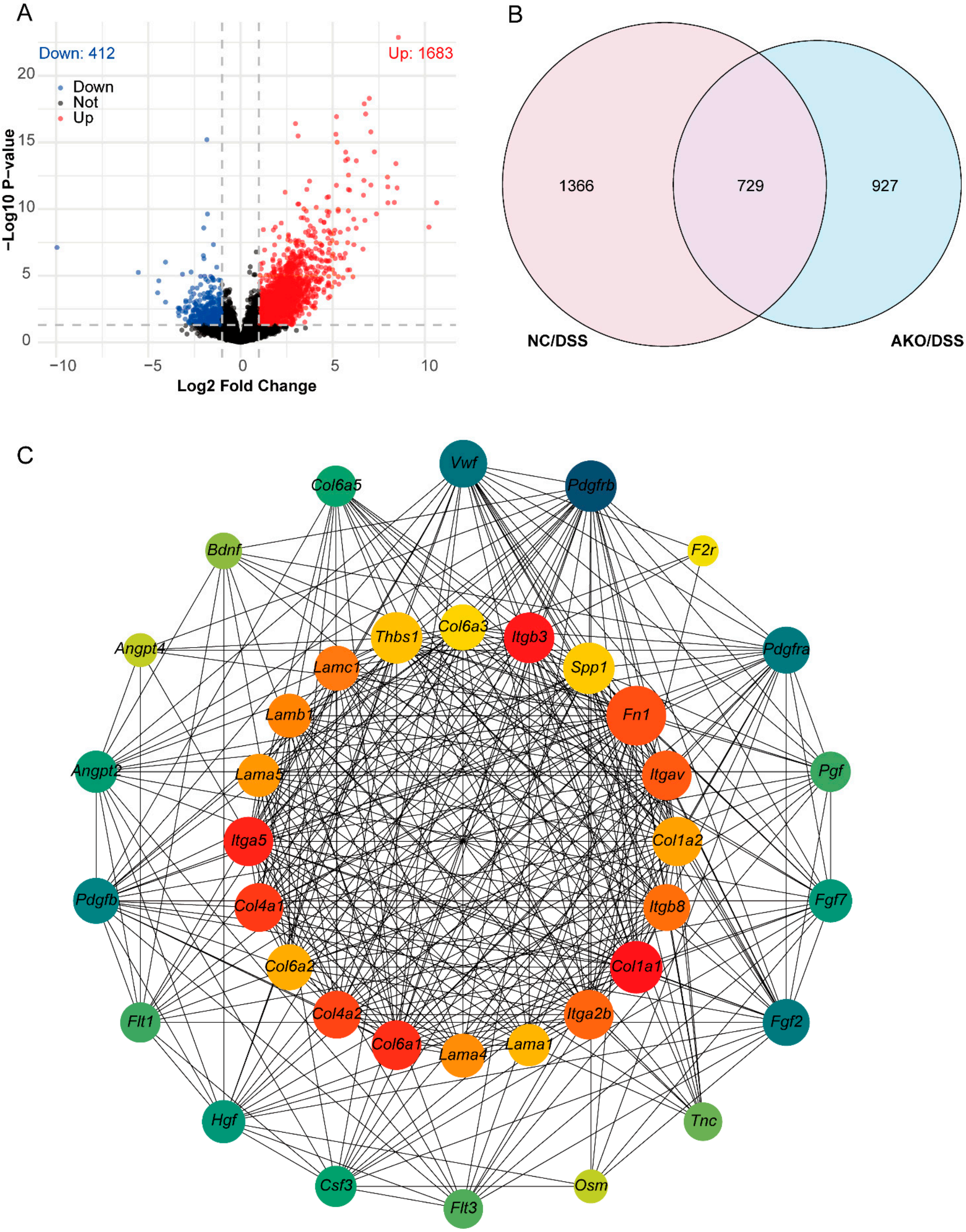

Figure S1. Transcriptomic profiling and protein-protein interaction analysis of DEGs. (A) Volcano plot of DEGs between DSS and NC groups. (B) Venn diagram of DEGs identified in the AKO vs. DSS and

DSS vs. NC comparisons. (C) Protein-protein interaction (PPI) network of key genes enriched in the PI3K/Akt signaling pathway.

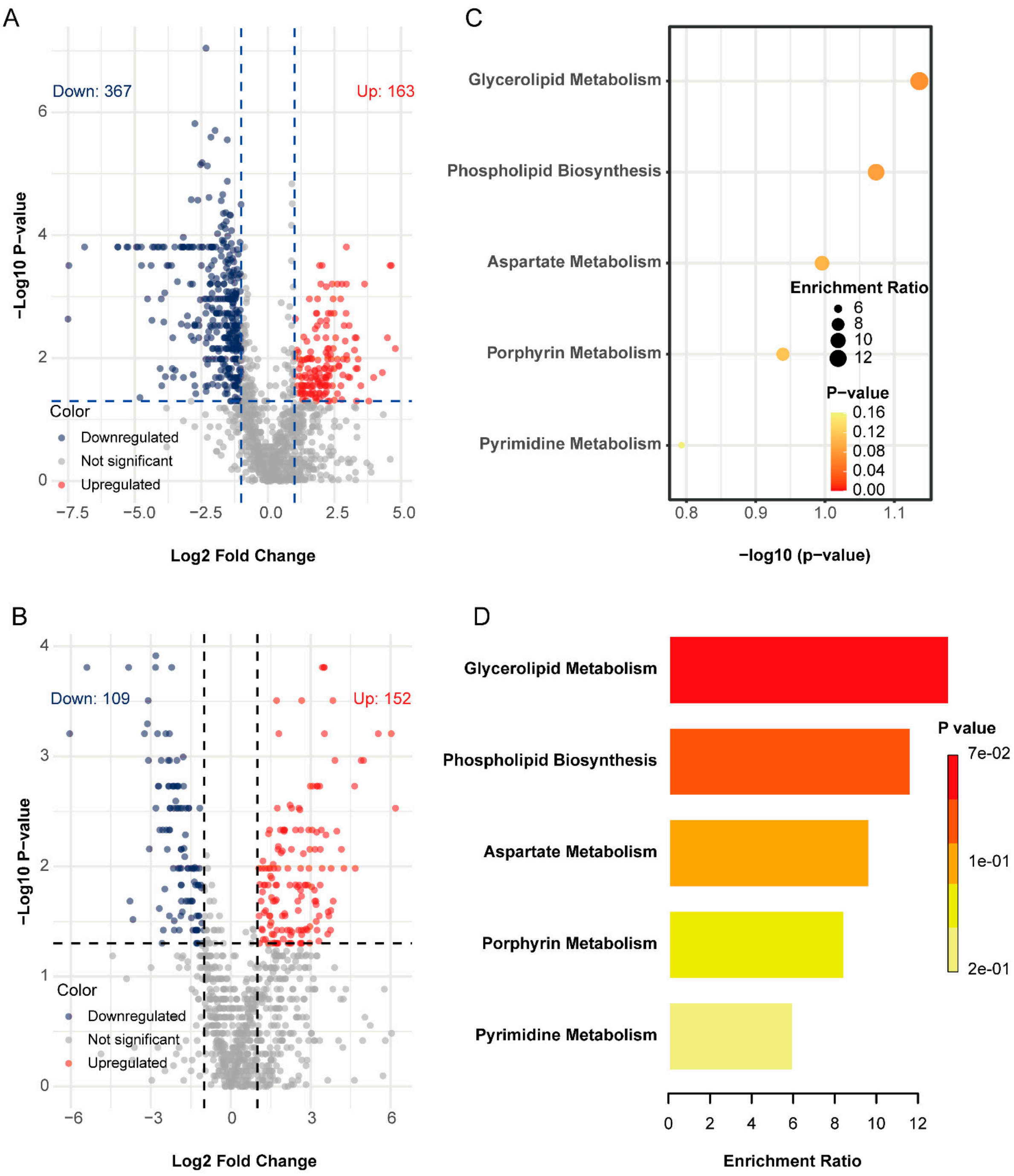

Figure S2. Metabolomic profiling and pathway enrichment analysis of differential metabolites. The volcano plot of significantly different metabolites between DSS and NC groups in (A) positive ion mode and (B) negative ion mode. (C) Bubble plot of differential metabolite enrichment analysis. (D) Bar plot of differential metabolite enrichment analysis.

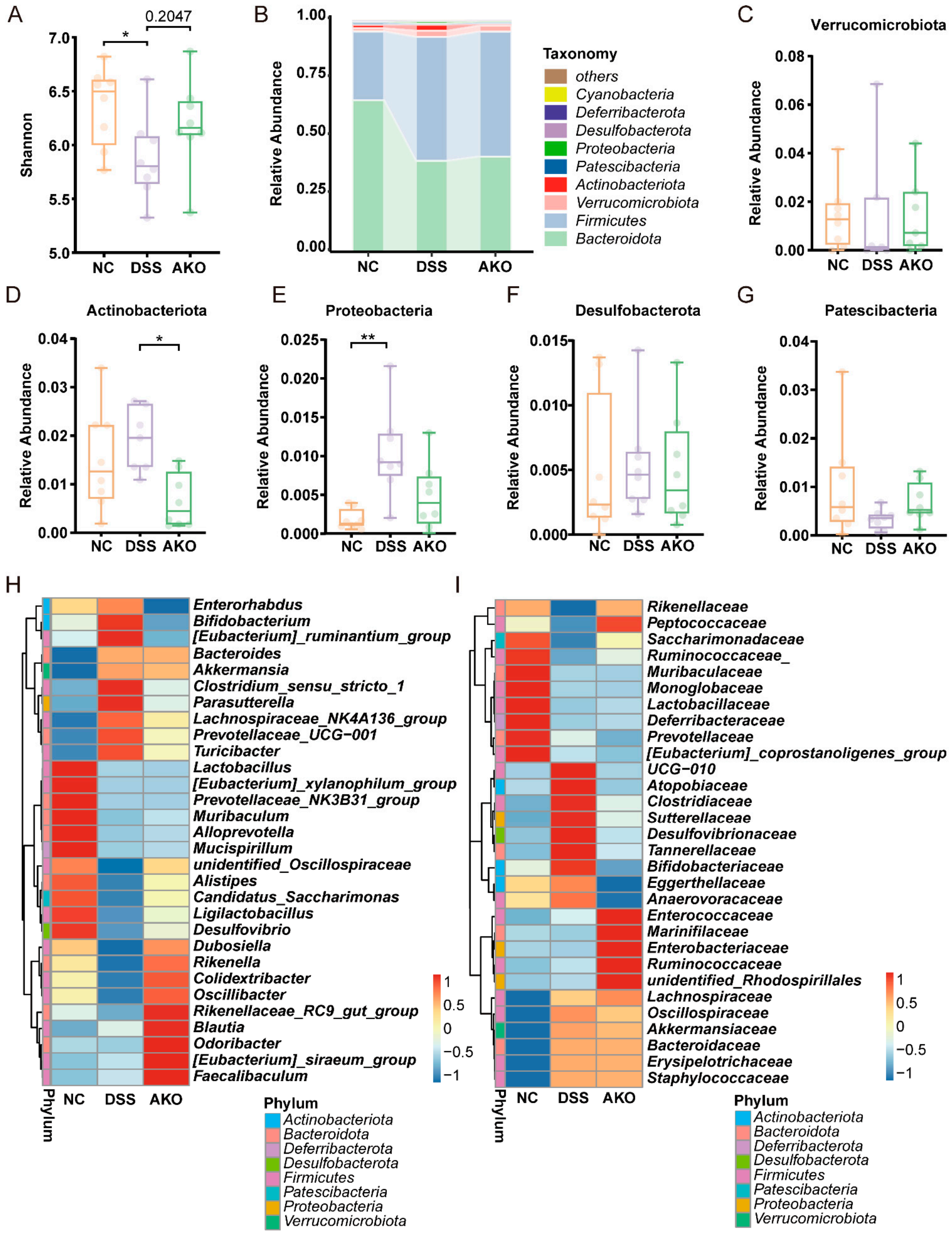

Figure S3. 16S rRNA analysis of gut microbial community composition. (A) Alpha diversity (Shannon index). (B) Gut microbiota composition at

the phylum level. (C-G) Relative abundance of (C)Verrucomicrobiota, (D)Actinobacteriota, (E)Proteobacteria, (F) Desulfobacterota, and (G) Patescibacteria. (H, I) Heatmaps of differentially abundant bacterial taxa at the family (H) and genus (I) levels

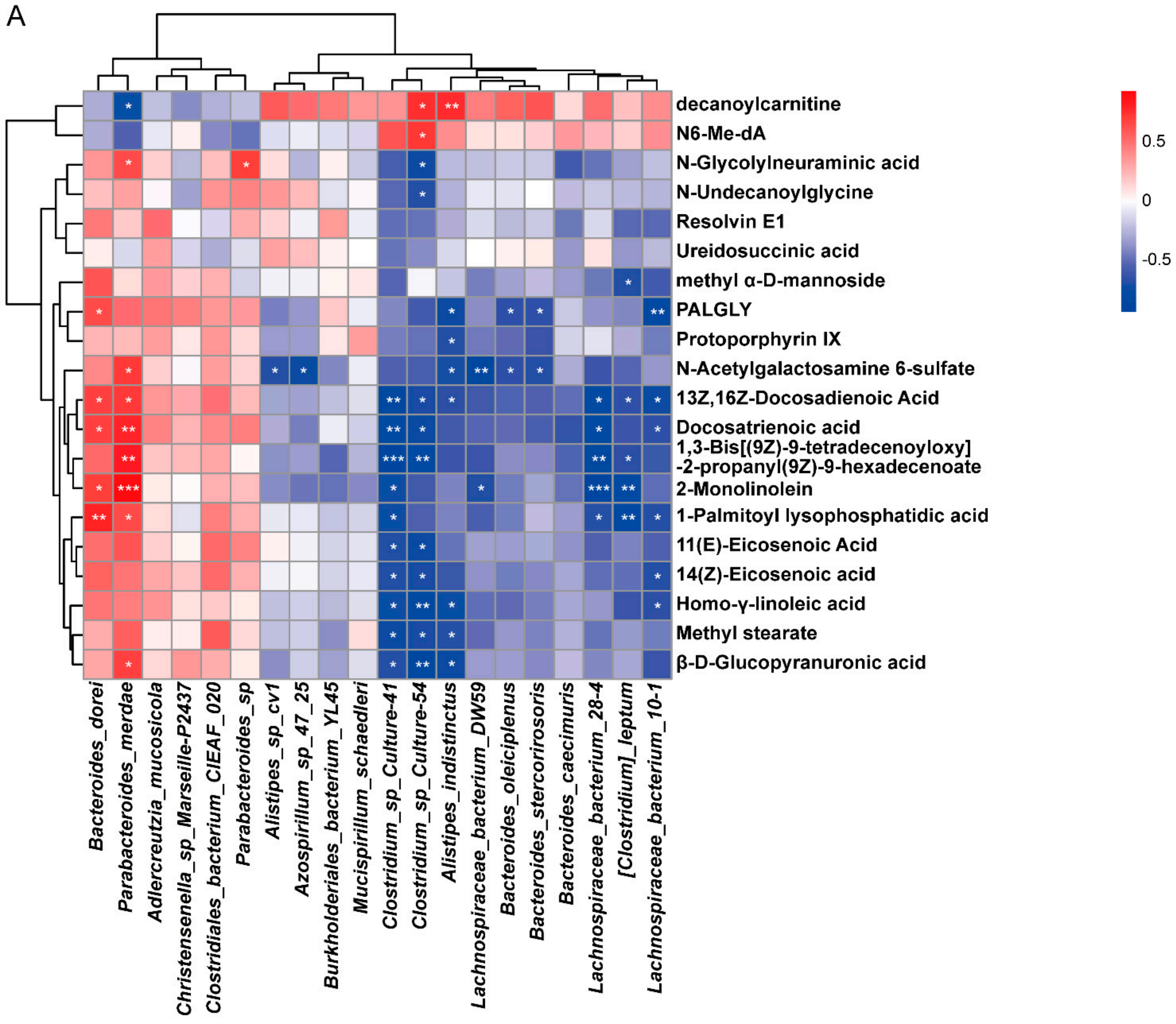

Figure S4. (A) Spearman correlation heatmap between the top 20 bacterial species and differential metabolites, *P < 0.05, **P < 0.01, ***P < 0.001.

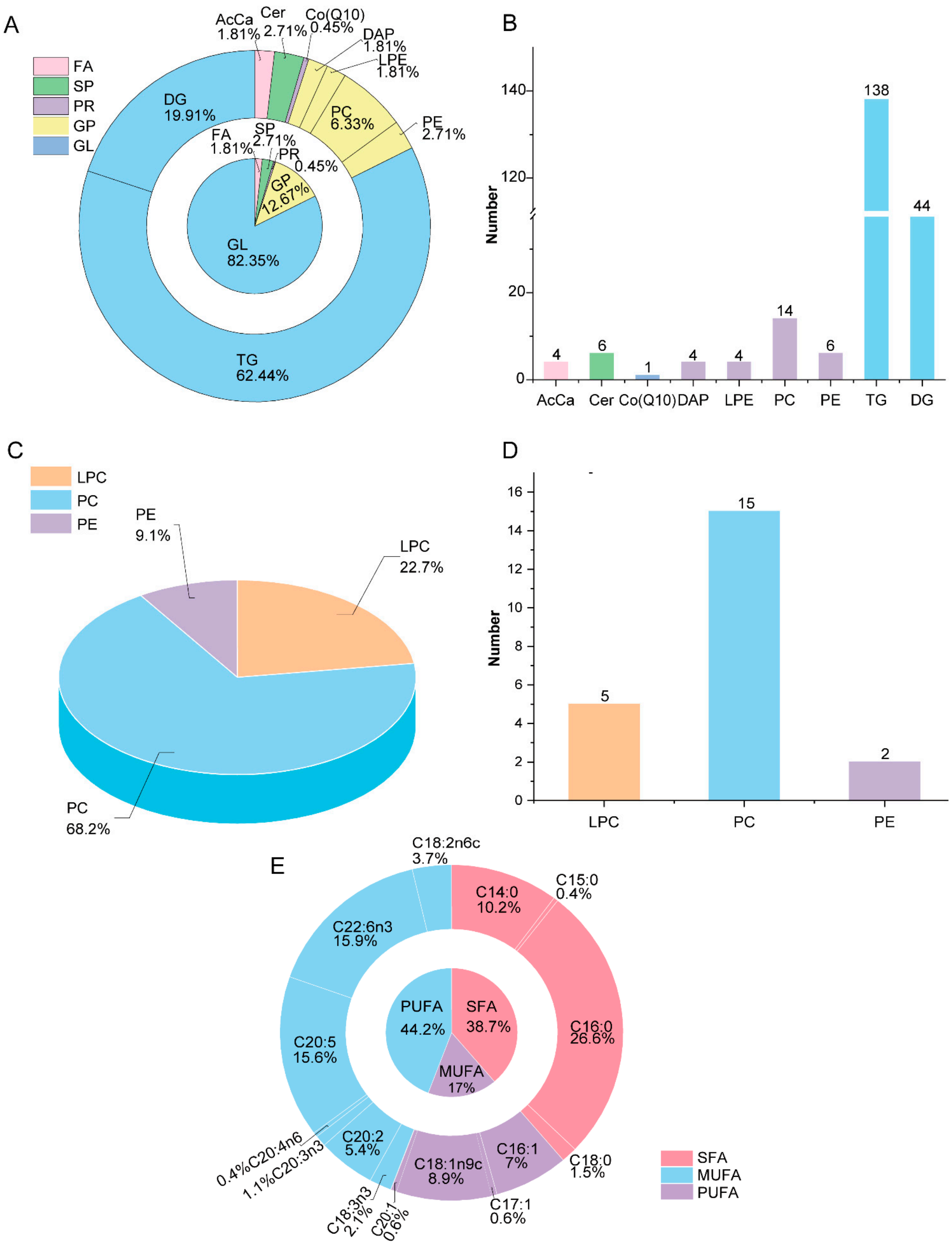

Figure S5. Composition of AKO. (A) Proportions of major lipid classes and subclasses detected in positive ion mode. (B) Numbers of major lipid classes and subclasses detected in positive ion mode. (C) Proportions of major lipid classes and subclasses in negative ion mode (D) Numbers of lipid subclasses detected in negative ion mode. (E) Composition of fatty acids in AKO.
